# Differential expression of immunity-related genes in larval *Manduca sexta* tissues in response to gut and systemic infection

**DOI:** 10.3389/fcimb.2023.1258142

**Published:** 2023-10-11

**Authors:** Yvette M. von Bredow, Petra Prochazkova, Jiri Dvorak, Frantisek Skanta, Tina E. Trenczek, Martin Bilej, Christoph-Rüdiger von Bredow

**Affiliations:** ^1^ Institute of Zoology and Developmental Biology, Justus-Liebig-University Gießen, Gießen, Germany; ^2^ Laboratory of Cellular and Molecular Immunology, Institute of Microbiology of the Czech Academy of Sciences, Prague, Czechia; ^3^ Applied Zoology, Department of Biology, Technische Universität Dresden, Dresden, Germany

**Keywords:** insect immunity, insect midgut, gut immunity, *Bacillus thuringiensis*, lysozyme, hematopoietic organ, comparative immune response

## Abstract

**Introduction:**

The midgut epithelium functions as tissue for nutrient uptake as well as physical barrier against pathogens. Additionally, it responds to pathogen contact by production and release of various factors including antimicrobial peptides, similar to the systemic innate immune response. However, if such a response is restricted to a local stimulus or if it appears in response to a systemic infection, too is a rather underexplored topic in insect immunity. We addressed the role of the midgut and the role of systemic immune tissues in the defense against gut-borne and systemic infections, respectively.

**Methods:**

*Manduca sexta* larvae were challenged with DAP-type peptidoglycan bacteria – *Bacillus thuringiensis* for local gut infection and *Escherichia coli* for systemic stimulation. We compared the immune response to both infection models by measuring mRNA levels of four selected immunity-related genes in midgut, fat body, hematopoietic organs (HOs), and hemocytes, and determined hemolymph antimicrobial activity. Hemocytes and HOs were tested for presence and distribution of lysozyme mRNA and protein.

**Results:**

The midgut and circulating hemocytes exhibited a significantly increased level of lysozyme mRNA in response to gut infection but did not significantly alter expression in response to a systemic infection. Conversely, fat body and HOs responded to both infection models by altered mRNA levels of at least one gene monitored. Most, but not all hemocytes and HO cells contain lysozyme mRNA and protein.

**Discussion:**

These data suggest that the gut recruits immune-related tissues in response to gut infection whereas systemic infections do not induce a response in the midgut. The experimental approach implies a skewed cross-talk: An intestinal infection triggers immune activity in systemic immune organs, while a systemic infection does not elicit any or only a restricted immune response in the midgut. The HOs, which form and release hemocytes in larval *M. sexta*, i) synthesize lysozyme, and ii) respond to immune challenges by increased immune gene expression. These findings strongly suggest that they not only provide phagocytes for the cellular immune response but also synthesize humoral immune components.

## 1 Introduction

The insect gut plays an outstanding role in controlling microbes that may colonize the gut by food uptake and forms, together with the integument, the first line of defense against pathogens (Siva-Jothy et al., 2005; Garcia-Garcia et al., 2013; Wu et al., 2016; Keehnen et al., 2017). In insects, the foregut and the hindgut are lined by a thin cuticle while the gut content is often physically separated from the midgut epithelium by a chitinous peritrophic membrane (Terra et al., 2019). Some pathogens, however, can penetrate the peritrophic membrane and the midgut epithelium (Vodovar et al., 2005). An infection is defined as the invasion and subsequent propagation of microorganisms in a living host (Thomson and Smith, 1994; Dorland, 2011). For clarity, we will use the terms “gut infection” for spatially restricted contact between potential pathogens and the gut epithelium, and “systemic infection” for physical contact between potential pathogens and multiple organs residing in the hemocoel. In the early stage of a gut infection, the host organism mounts a local immune response in the gut, but should also prepare for a potential septic event e.g. by a systemic upregulation of immune-related genes. Setting the organism to a state of alert might therefore be a prerequisite to surviving an otherwise lethal infection of the hemocoel caused by local infection with pathogens impairing the gut integrity such as *Bacillus thuringiensis* (Sampson and Gooday, 1998; Caccia et al., 2016). The fat body – the main source of antimicrobial peptides (Trenczek and Faye, 1988) – the hemocytes, and probably the hematopoietic tissues may therefore react to a gut-borne infection by increased synthesis of immune-related genes even before bacteremia occurs. While the gut must provide an environment suitable for commensals and symbionts, pathogens must be sensed and an immune response mounted if necessary. The immune response in the insect gut is therefore tightly regulated (Ryu et al., 2008; Paredes et al., 2011). Pathogens that are ingested during food uptake are sensed by pathogen recognition receptors (PRRs) of the gut which bind to evolutionary conserved microbe-associated molecular patterns (MAMPs). At the same time, however, gut cells ignore harmless microbes and tolerate symbiotic microbes which share many of the MAMPs with pathogens, and by this an immune overreaction is avoided (Engel and Moran, 2013; Zeng et al., 2022; Zhang et al., 2022).

While the gut has historically been thought to be solely responsible for the breakdown and resorption of nutrients, a new view emerged with more recent research. The intestine of humans, for example, is now seen as a major checkpoint for immunity, and the composition of its microbiota and its cross-talk with the immune system are responsible for an organism’s health in general (Abraham and Medzhitov, 2011). The imbalance of the intestinal microbiota and immune response can lead to local and systemic diseases (Jiao et al., 2020). Therefore, the intestine-immune system-internal organ axis is a highly important pathway for an organism’s health and integrity. Many mechanisms of the innate immune system are evolutionarily conserved. One example is the general role of PRRs of the peptidoglycan recognition protein type (PGRPs) which play vital roles in microbe recognition and the immune response throughout the metazoan kingdom (Dziarski and Gupta, 2006; Dziarski and Gupta, 2010). PGRP-1 of *M. sexta* is capable of binding to diaminopimelic acid-type peptidoglycan (DAP-type PGN; Sumathipala and Jiang, 2010) present in *B. thuringiensis* (Kingan and Ensign, 1968) and gram-negative bacteria such as *E. coli* cell walls, and activates the prophenoloxidase cascade (Yu et al., 2002; Sumathipala and Jiang, 2010). Discrimination of pathogens or an otherwise unbalanced gut microbiota is thought to take place through a combinatorial activity of mechanisms either eliciting or suppressing an immune response. Activation of an immune response comprises sensing of “honest signals” secreted by the pathogenic bacteria (e.g. uracil, Lee et al., 2013) as well as binding to commonly occurring MAMPs such as peptidoglycan (PGN), lipopolysaccharide (LPS), or lipoteichoic acid (LTA) by specific PRRs (reviewed e.g. in Gillespie et al., 1997; Tsakas and Marmaras, 2010). A variety of different receptors tightly regulates the immune response by either activating or repressing cues. In *Drosophila melanogaster*, for example, a distinct PGRP cleaves bacterial PGN into smaller fragments with lower immunogenic potential than uncleaved PGN. This activity results in a reduced immune response (Paredes et al., 2011; Costechareyre et al., 2016).

A battery of immune-related genes is expressed in the midgut of *Manduca sexta*, including proteins involved in sensing pathogens and proteins with distinct antimicrobial activity (Russell and Dunn, 1991; Russell and Dunn, 1996; Pauchet et al., 2010). For example, the gut of *M. sexta* responds to a gut infection with a locally restricted upregulation of nitric oxide synthetase (NOS) expression (Eleftherianos et al., 2009).

A piece of evidence for the involvement of tissues other than the midgut itself in gut immunity was given by Rupp and Spence (1985) for *M. sexta.* They found a protein (M13, alias scolexin) to appear in the hemolymph of *B. thuringiensis* toxin-fed larvae. This protein is probably involved in hemolymph coagulation (Minninck et al., 1986; Kyriakides et al., 1993). We hypothesize that in *M. sexta* an enteric infection may induce a systemic immune response and a systemic infection may induce an immune response in the gut epithelium, as has been shown for other insect species (Das De et al., 2018; Meraj et al., 2021). Further support of an expected cross-talk between gut and systemic immune cells come from a study by McMillan and Adamo (2020), where systemic inoculation of a mixture of heat-killed bacteria and fungi increased AMP mRNA synthesis in the gut of *M. sexta*. We tested our hypothesis with a comparative approach using live microbes and present a first study testing both the reaction of non-midgut tissues on a gut-mediated infection and the reaction of the midgut on a systemic infection in *M. sexta* larvae. As a basis for further studies, we used selected immune parameters as indicators for immune system activation.

## 2 Methods

### 2.1 Insects

*Manduca sexta* specimens were obtained from the laboratory stock of the Institute of General and Applied Zoology, Justus-Liebig-University, Gießen, Germany. Larvae were kept individually at 24°C under long-day conditions 16:8 h (light:dark), controlled regularly for health status (growth, feces, melanization), and only healthy individuals were used for experiments. Animals were fed with an antibiotic-free artificial diet, modified after Yamamoto (1969): Whole grain flour 5.27% (w/v), soy meal 5.70% (w/v), agar-agar Kobe I 2.55% (w/v), casein 1.40% (w/v), plant oil 0.16% (w/v), ascorbic acid 30.02 mM, methylparabene 11.54 mM, sorbic acid 15.66 mM, formaldehyde 5.33 mM, CaCO^3^ 5.28 mM, NaCl 9.03 mM, nicotinic acid 97.47 µM, riboflavin 15.94 µM, folic acid 6.34 µM, pyridoxine 16.55 µM, thiamine hydrochloride 9.31 µM and biotin 9.82 µM.

### 2.2 Bacteria

*Escherichia coli* K12 culture was obtained from the laboratory stock of the Institute of Microbiology, Academy of Science of the Czech Republic, Prague.

*Bacillus thuringiensis* ssp. *kurstaki* and *E. coli* K12 strain D31 (Monner et al., 1971) were obtained from laboratory stocks of the Institute of General Zoology and Developmental Biology, Justus-Liebig-University, Gießen, Germany.

All bacteria were grown in liquid Standard Nutrient Broth I (C. Roth GmbH, Karlsruhe, Germany) prepared according to manufacturer´s protocol.

*B. thuringiensis* was grown in liquid culture on a rotary shaker at 180 rpm at room temperature for approx. 15 days until spore density reached approx. 50%. Sporulation was controlled by phase contrast light microscopy. After reaching 50% sporulated bacteria, liquid cultures were stored at 4°C and used within one week for infection experiments.

### 2.3 Immune stimulation

Immune stimulations were conducted 15 hours prior to RNA isolation. Each treatment was performed three times in independent cohorts of *n* = 4 L5d1 larvae, i.e. between 24 and 48 hours after moulting to L5. Tissues of each cohort were pooled before RNA isolation.

#### 2.3.1 *E. coli* K12-injection

A fresh overnight culture of *E. coli* K12 was rinsed twice with sterile saline (155 mM NaCl) and diluted to 2×10^8^/ml bacteria in saline. 2×10^6^ bacteria (10 µl suspension) per gram body weight were injected ventrolaterally left at the border between abdominal segments five and six. Larvae injected with bacteria-free saline served as control.

#### 2.3.2 *Bacillus thuringiensis* feeding

Food blocks of 1 cm^3^ size were soaked in a suspension of 50% sporulated *B. thuringiensis* ssp. *kurstaki* in water and air dried in a laminar flow bench. Each cube contained 2.5×10^6^ spores. *M. sexta* larvae exhibit interval feeding behavior when kept on an artificial diet (Reynolds et al., 1986). Hence, to ensure immediate uptake of the food blocks, the larvae were starved for 12 hours before feeding. The larvae never consumed whole food blocks before they stopped food uptake. Therefore, we calculated the amount of ingested spores by measuring the consumed diet surface area. Mean spore uptake was 8.8×10^4^ per animal, SD 3.9×10^4^ (range: 5×10^4^ to 1.9×10^5^). As a control, equally starved animals were fed artificial diet cubes soaked with an equal amount of sterile water.

### 2.4 Determination of growth rate

The growth rate of the larvae during the period of immune stimulation was calculated with the following formula: Growth rate = (body weight_t1_ – body weight_t0_) × body weight_t0_^-1^; with t_0_ = time point of immune challenge (injection or feeding) and t_1_ = t_0_ + 15 hours.

### 2.5 Determination of immune status

From each animal analyzed in this study, 50 µl hemolymph was collected at the time point of RNA isolation, frozen immediately, and stored at -80°C.

Lytic activity was quantified by radial diffusion agar assay as outlined by Mohrig and Messner (1968). Activity is expressed in µg/ml hen egg white lysozyme equivalents (HEWLE).

Additionally, bactericidal/bacteriostatic activity assay against live *E. coli* K12 strain D31 was determined by radial diffusion agar assays as described by Hultmark et al. (1982). Serially diluted Gentamicin served as a reference antibiotic. Antimicrobial activity of the hemolymph is expressed in µg/ml Gentamicin equivalents (GE).

### 2.6 Statistical analysis

All data were tested for normal distribution by Kolmogorov-Smirnov-test. Non-normal distributed data (growth rate, lytic activity, and antimicrobial activity) were tested for significance (*p* ≤ 0.05) by the asymptotic Mann-Whitney-*U*-test (R V. 3.0.3, R Development Core Team, 2008; http://www.R-project.org; package *coins*, Hothorn et al., 2008, function *wilcox_test*) and data presented as median (Mdn) and interquartile range (IQR). Box-and-whiskers-plots with median (horizontal line), upper and lower quartile (boundaries), and 1.5-fold interquartile range (whiskers) were created with R V. 3.0.3. Normally distributed data (real-time PCR data) were evaluated for significance by two way ANOVA with *post hoc* Tukey´s multiple comparison (GraphPad Prism V. 5.0, GraphPad Software, San Diego, California, USA). Differences were considered significant with *p* ≤ 0.05. All values are presented as mean ± standard deviation. Bar plots of real-time PCR data were created with GraphPad Prism V. 5.0.

### 2.7 Tissue preparation

Animals were cleaned with water, surface disinfected with 70% (v/v) ethanol, and chilled on ice for sedation. Midgut and fat body tissues were dissected with fine forceps and scissors (contralaterally to injection sites for *E. coli* K12-injected and saline-injected control) at three regions of each animal: anterior ventrolateral (abdomen segment 1), middle part ventrolateral (abdomen segment 4/5), posterior ventrolateral (abdomen segment 6/7) and washed twice with calcium and magnesium-free *Manduca sexta* saline (MS-, Willot et al., 1994). Hematopoietic organs (HO) were removed completely (four HOs per animal) and washed twice with MS-. Hemolymph from each group was collected in 6 ml ice cold anticoagulant saline (AC saline, Willot et al., 1994), sedimented at 500 x *g* for 10 min at 4°C, and the resulting hemocyte pellet was used for RNA isolation.

### 2.8 RNA isolation and cDNA synthesis

Whole RNA from fat body, hematopoietic organ, midgut, and hemocytes was isolated with an RNeasy Mini RNA purification kit (Qiagen, Hilden, Germany). Pooled tissue from four animals was used for each isolation. The RNA quality, purity, and concentration were evaluated spectrophotometrically with a NanoDrop 1000 (Thermo Fisher, Waltham, USA) and denaturing RNA electrophoresis. Prior to reverse transcription, the contaminating gDNA was removed by DNAse I digestion (DNAse I RNAse free, Thermo Fisher, Waltham, USA). 550 ng purified RNA of each sample was transcribed to cDNA with SuperScript III Reverse Transcriptase (Life Technologies, Carlsbad, USA) using oligo(dT)20 primers.

### 2.9 Quantitative real-time PCR

Quantitative PCR analysis was performed to determine changes in the mRNA levels coding for the immune molecules immulectin-3, scolexin A, PGRP 1A, and lysozyme. Each reaction was prepared as follows: 12.5 µl iQ^TM^ SYBR^®^ Green Supermix (Bio-Rad Laboratories Inc., Hercules, USA), 1 µl of each forward and reverse primers (**Table 1**), and 4 µl of cDNA diluted in RNase-free water (1:20). PCR was done using a CFX96Touch Real-time PCR Detection System (Bio-Rad Laboratories Inc., Hercules, USA). The amplification protocol was as follows: 3 min at 95°C followed by 40 cycles of 94°C for 30 s, 57°C for 40 s, and 72°C for 70 s. The temperature was then gradually increased to 95°C to obtain melting curves of the amplified fragments, which confirmed the specificity and uniformity of the PCR products. Evaluation of amplification efficiency was performed by dilution series of cDNA forming the standard curve. The expression levels were calculated with 2^-ΔΔCq^ method (Livak and Schmittgen, 2001). To select suitable housekeeping genes, the gene expression stability was assessed using RefFinder (https://www.heartcure.com.au/reffinder/). Two reference genes (ribosomal protein S3, RPS3; translation elongation factor 1 alpha, EF1A) were selected as the most stable internal controls for the normalization of the expression of other genes. Non-template controls were included in each experiment. All parameters were measured in duplicates. The fold change in the mRNA level was related to the change in the settled controls. For the treatments “*B. thuringiensis*-fed” untreated L5d2 larvae served as a control group. For the treatment “*E. coli* K12-injected” the “saline-injected” group was used as a control, which resembles the injection procedure without bacteria. The results were expressed as the mean ± SD of the values obtained in three independent experiments.

**Table 1:** Specific primers used in qPCR and for mRNA probe synthesis.

gene	forward primer	reverse primer	GenBank Acc. No.
immulectin-3 (IML-3)	GAAGAAGCTGGCTATGCGAA	TGCGCACTACACTAATGACG	AY768811.1
scolexin A	ATACGCAGTTCGGAGTTTCG	CAGACGGGTCCTATGGAGAA	AF087004.1
PGRP 1A	ATCTTCGTTCCTGATTGGCG	CAGCAGGGCTTTGATAGCAT	AF413068.1
lysozyme	GTGGAGAATGAGAGCAGCAG	GTACCACGCTTGGAACTTGT	S71028
RP S3	GTCGGTGACGGAGTTTTCAA	CTTTCTCAGCGTACAGCTCC	U12708.1
EF 1A	CTTCACAGCTCAGGTCATCG	GAAGGACTCCACACACATGG	AF234571.1
lysozyme *	GAAAATTTAGGTGACACTATAGAAGNGTCTTGTTGCCGACGACTTCTC	GAAATTAATACGACTCACTATAGGGAGATTGTGGCGTTTGTATATCTTCTTG	S71028
PGRP-1A *	GAAAATTTAGGTGACACTATAGAAGNGACCTGGCTGCGATACCGACGAC	GAAATTAATACGACTCACTATAGGGAGAGCGATGGCCCACGACTTTGTAAT	AF413068.1

* = oligonucleotides used for the synthesis of RNA probes containing T7 and SP6 promoters. Underlined nucleotides depict gene-specific sequences.

All specific primers were designed and tested for suitability with the primer3 online tool (http://primer3.ut.ee/; Rozen and Skaletsky, 2000).

### 2.10 *In situ* mRNA hybridization

Digoxigenin-labeled RNA probes against lysozyme were synthesized from a 1301 bp long DNA template, amplified by PCR (initial denaturation 94°C 3 min; 40 repeats of 94°C 25 s, 60°C 30 s, 72°C 45 s; and final extension of 72°C 7 min) with gene-specific forward and reverse primers and added sequences for the SP6 or T7 promoters, respectively (**Table 1**). Digoxigenin-labeled RNA probes were synthesized with the DIG RNA labeling kit SP6/T7 (Hoffmann-La Roche AG, Basel, Switzerland) according to the manufacturer’s protocol.

Standard *in situ* hybridization (ISH) of whole hematopoietic organs and hemocyte monolayers was performed as described elsewhere (hematopoietic organs: von Bredow et al., 2020; hemocytes: von Bredow et al., 2021) with 2 ng/µl riboprobe. Bound riboprobes were visualized using alkaline phosphatase-conjugated sheep anti-DIG Fab (Hoffmann-La Roche AG, Basel, Switzerland) and chromogenic substrate (4 mM nitroblue tetrazolium chloride, 5.5 mM 5-bromo-4-chloro-3-indolyl phosphate).

For fluorescence *in situ* hybridization (FISH), the riboprobe concentration was 5 ng/µl. Instead of a chromogenic reaction, the sheep anti-DIG Fab AP was labeled with biotin-labeled goat anti-sheep immune serum (Vector Laboratories, Burlingame, USA) followed by signal enhancement with the Vectastain ABC Kit Standard (Vector Laboratories, Burlingame, USA) and the TSA Amplification Renaissance Kit (PerkinElmer Inc., Waltham, USA). Nuclei were stained with 360 nM 4′,6-diamidino-2-phenylindole (DAPI). The specimens were mounted with Fluoromount G (Southern Biotech, Birmingham, USA) and analyzed with a Leica TCS SP2 confocal laser scanning microscope (Leica Microsystems GmbH, Wetzlar, Germany).

For both lysozyme ISH and FISH, a digoxigenin-labeled sense riboprobe specific for a 319 base sequence of PGRP-1A mRNA was used as negative control since we were not able to synthesize a sufficient amount of digoxigenin-labeled lysozyme sense riboprobe. Control reactions were performed for each hybridization i) by incubation with digoxigenin-labeled PGRP-1A sense riboprobes to rule out non-specific binding of riboprobes, and ii) by omitting any riboprobes to rule out non-specific antibody binding, endogenous alkaline phosphatase activity (ISH), and non-specific fluorochrome adhesion or autofluorescence (FISH).

### 2.11 Immunofluorescence

Hematopoietic organs and hemocyte monolayers were immunolabeled with rabbit-anti-*Hyalophora cecropia* lysozyme immune serum (Trenczek and Faye, 1988), diluted 1:1000 in 3% BSA in TBS. Hematopoietic organs were additionally labeled with mouse monoclonal antibody MS75 (Beetz et al., 2004). Nuclei were stained with 360 nM DAPI. For a detailed immunolabeling protocol, please see von Bredow et al., 2020. The slides were mounted with Fluoromount G^®^ and analyzed by confocal laser scanning microscopy.

## 3 Results

### 3.1 Effect of gut infection and bacteria injection on the larval growth rate

**Figure 1:**
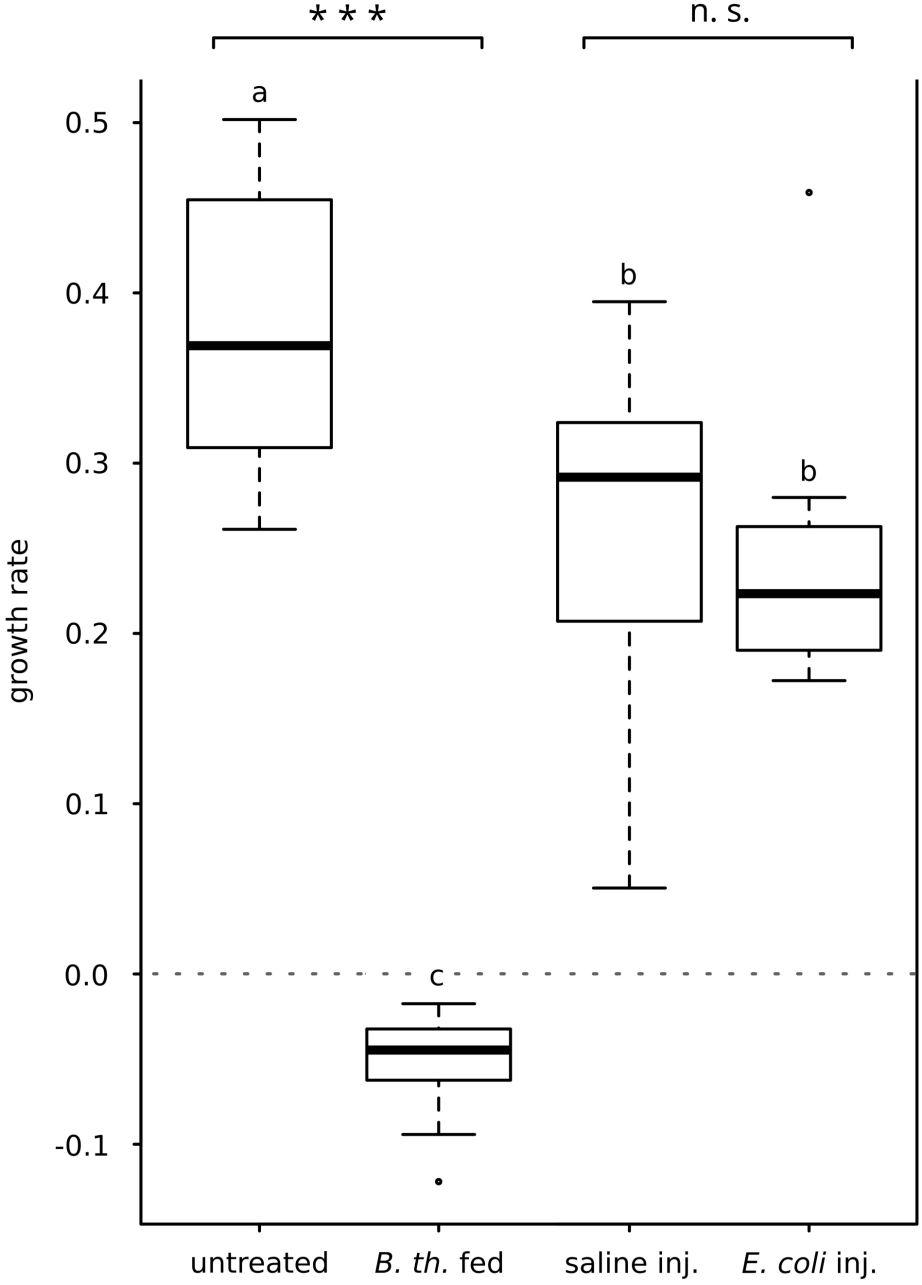
Effect of *E*. *coli* K12-injection and *B*. *thuringiensis* ssp. *kurstaki* oral uptake on larval growth rate. *E*. *coli* K12-injection did not alter the growth rate significantly compared to the saline-injected animals. *B*. *thuringiensis* infection greatly decreased the growth rate, differing significantly from the untreated control group. Bars represent median values, borders represent upper and lower quartiles, whiskers depict 1.5-fold IQR, black circles depict outliers. Significant differences were calculated by Mann-Whitney-*U*-test. Significant differences between immune challenge and respective control are depicted by asterisks (*** *p* ≤ 0.001; n.s., not significant). Overall significant differences between all treatments (*p* ≤ 0.05) are marked by different lowercase letters (a, b, c). *B. th.* fed, *Bacillus thuringiensis* ssp. kurstaki-fed; saline inj., saline-injected; *E. coli* inj., *E. coli* K12-injected.

The increase in body weight of larvae served as an indicator of the overall health status. Injection of *E. coli* K12 did not alter the growth rate significantly when compared to saline-injected control group (growth rate *E. coli*-injected Mdn 0.22, IQR 0.06; growth rate saline-injected Mdn 0.29, IQR 0.11; *p* = 0.273, Mann-Whitney-*U*-test; **Figure 1**). Infection with *B. thuringiensis* caused a loss of body weight within 15 hours after the first pathogen uptake, therefore resulting in negative growth rates differing significantly from untreated controls (growth rate *B. thuringiensis*-fed Mdn = -0.04, IQR 0.03; growth rate untreated Mdn 0.37, IQR 0.13; *p* = 0.000, Mann-Whitney-*U*-test; **Figure 1**).

### 3.2 Effect of gut infection and bacteria injection on the lysozyme-activity of the hemolymph

**Figure 2:**
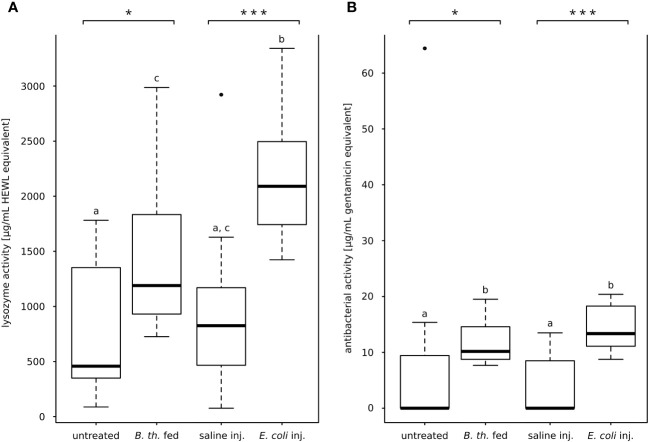
Lytic activity and anti-*E. coli* K12 D31-activity of hemolymph. **(A)** Lysis of dead *M. luteus*, lytic activity in HEWL-equivalents. Both oral *B*. *thuringiensis* infection and injection of *E*. *coli* K12 increased the lytic activity of the hemolymph significantly. **(B)** Zone of inhibition assay against *E*. *coli* K12 D31, antibacterial activity in gentamicin equivalents. *E*. *coli K12-*injection and oral *B*. *thuringiensis* infection increased significantly antibacterial activity compared to the respective control. Bars represent median values, borders represent upper and lower quartiles, whiskers depict 1.5-fold IQR, black circles depict outliers. Significant differences were calculated by Mann-Whitney-*U*-test Significant differences between immune challenge and respective control are depicted by asterisks (* *p* ≤ 0.05; *** *p* ≤ 0.001). Different lowercase letters (a, b, c) depict overall significant differences between all treatments (*p* ≤ 0.05). *B. th.* fed, *Bacillus thuringiensis ssp. kurstaki-*fed; saline inj., saline-injected; *E. coli* inj., *E. coli* K12-injected.

Lytic activity of the hemolymph was observed for all treatment and control groups (**Figure 2A**). Both injection of *E. coli* K12 and feeding *B. thuringiensis* resulted in increased lysozyme activity in the hemolymph compared to the respective control. The lytic activity (µg/ml HEWLE) of *E. coli* K12-injected animals was significantly higher compared to saline-injected controls (*E. coli-*injected, Mdn 2090 µg/ml HEWLE, IQR 706; saline-injected Mdn 825 µg/ml HEWLE, IQR 582; with *p* = 0.000, Mann-Whitney-*U*-test; **Figure 2A**). In *B. thuringiensis-*fed animals, the lytic activity was also significantly higher than in untreated controls (*B. thuringiensis*-fed, Mdn 1189 µg/ml HEWLE, IQR 789; untreated, 458 µg/ml HEWLE, IQR 929; with *p* = 0.016, Mann-Whitney-*U*-test; **Figure 2A**).

### 3.3 Effect of gut infection and bacteria injection on the antibacterial activity of the hemolymph

The hemolymph antibacterial activity against live *E. coli* K12 strain D31 was measured for each treatment. Most hemolymph samples of untreated animals and saline-injected animals did not inhibit bacterial growth (*n* = 7 of 12 for each control cohort). Conversely, inhibition was observed for all samples from both *E. coli* K12-injected and *B. thuringiensis*-fed animals (*n* = 12 of 12 for each treatment cohort) (**Figure 2B**). The antibacterial hemolymph activity was significantly increased in both bacterial treatments compared to the respective control (*E. coli* K12-injected Mdn. 13.36 µg/ml GE, IQR 7.06; saline-injected Mdn. 0.00 µg/ml GE, IQR 7.76; with *p* = 0.000, Mann-Whitney-*U*-test; *B. thuringiensis*-fed Mdn. 10.17 µg/ml GE, IQR 5.45; untreated Mdn. 0.00 µg/ml GE, IQR 9.37; with *p* = 0.013, Mann-Whitney-*U*-test; **Figure 2B**).

### 3.4 Bacteria recovery from hemolymph after experimental infection

Animals treated the same way as for the qPCR experiments were tested for bacterial hemocoel invasion 15 hours after feeding *B. thuringiensis*, and survival of injected *E. coli* K12 in the hemocoel, respectively. Hemolymph samples were spread on nutrient agar plates and the number of colony-forming units (CFUs) was determined. Colonies occurred rarely in untreated samples or saline-injected animals. They most likely originated from contamination during the sampling procedure (**Supplementary Figure 1**).

Bacteria colonies occurred only seldom in samples of *B. thuringiensis-*infected animals. The morphology of the colonies resembled that observed for *B. thuringiensis* colonies in one case (data not shown) with 40.4 CFU/µl recovered from the hemolymph. This was the only case (of four in total) that indicates an invasion of microorganisms from the gut after experimental gut infection (**Supplementary Figure 1**). A second sample exhibited a very low CFU count (0.2 CFU/µl), which is within the range of CFUs recovered in untreated controls (**Supplementary Figure 1**).

Since the extent of gut damage is likely proportional to the concentration of *B. thuringiensis* toxins ingested, we administered two additional cohorts (*n* = 4 animals each) with higher *B. thuringiensis* concentrations (5×10^6^ and 1×10^7^ spores per 1cm^3^ diet cube). No CFUs with the typical morphological appearance of *B. thuringiensis* occurred in any sample, indicating that only rarely (i.e. in one of twelve cases across all concentrations) an invasion of gut bacteria into the hemocoel appears within 15 hours after *B. thuringiensis* infection.

Fifteen hours after *E. coli* K12-injection CFUs resembling the morphology and size of *E. coli* K12 colonies were isolated from the hemolymph in various densities (0.8, 1.2, 3, and 55.2 CFU/µl) from *n* = 4 animals tested (**Supplementary Figure 1**). These data suggest that injected *E. coli* K12 were not completely cleared from the hemocoel within 15 hours, indicating a persisting experimental infection in the hemocoel.

### 3.5 Survey of health status and observations during dissection

During preparation, animals were examined for the following parameters: Body rigidity, melanotic capsules in the body cavity, and integrity of the peritrophic membrane of the gut.

Untreated controls, saline-injected controls, and *E. coli* K12-injected animals were turgescent whereas *B. thuringiensis-*fed animals were flabby and responded with convulsive muscular contractions to tactile stimuli. Melanotic capsules were present in the hemocoel of each *E. coli* K12-injected larva, but absent in untreated, saline-injected, and *B. thuringiensis-*fed animals. The peritrophic membrane of the gut was stable in untreated controls, saline-injected controls, and *E. coli* K12-injected animals, allowing the removal of the gut content by taking out the peritrophic membrane tube. However, in *B. thuringiensis-*fed animals the peritrophic membrane was fragile and disintegrated into small fragments during preparation.

### 3.6 Effect of gut infection and systemic infection on the mRNA level of immunity-related genes in hematopoietic organs, hemocytes, fat body, and midgut

**Figure 3:**
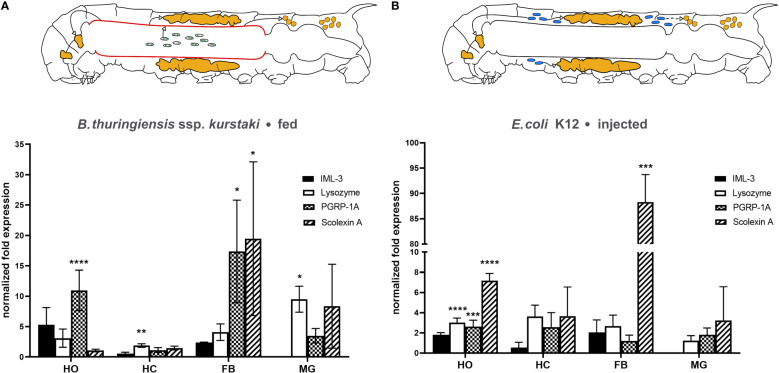
Changes in immune-related mRNA levels in response to **(A)** oral *B. thuringiensis* infection and **(B)** *E*. *coli* K12 injection in hematopoietic organs (HO), hemocytes (HC), fat body (FB), and midgut (MG). The caterpillar schematics summarize the overall effect of each infection model. The gut infection (depicted by sporulated bacteria in the gut lumen) affects each tissue observed (indicated by green arrows), including the midgut itself (red) **(A)**, the systemic infection (depicted by blue bacteria) affects the hematopoietic organs and the fat body. Bars depict mean fold change, whiskers depict standard deviation. Significant changes to the respective control are labeled by asterisks (* *p* ≤ 0.05; ** *p* ≤ 0.01, *** *p* ≤ 0.001, **** *p* ≤ 0.0001; two-way ANOVA with *post hoc* Tukey´s multiple comparison). The expression of the genes was normalized to EF1a and rps3 expression. Fold expression was calculated by the 2^-ΔΔCq^ method. See main text for details.

In response to *B. thuringiensis* infection each examined tissue increased expression of one or more immunity-related genes (**Figure 3A**). Hematopoietic organs exhibited highly significantly increased PGRP-1A mRNA levels (10.96 ± 3.34, *p* < 0.0001). Hemocytes responded by slightly but significantly increased lysozyme mRNA level (1.89 ± 0.29, *p* = 0.047) (**Figure 3A**). The levels of PGRP-1A (17.37 ± 8.45, *p* = 0.0317) and scolexin A mRNA (19.47 ± 12.65, *p* = 0.014) were significantly increased in the fat body (**Figure 3A**). In the midgut tissue, only lysozyme mRNA level was significantly increased (9.52 ± 2.14, *p* = 0.034) (**Figure 3A**). IML-3 mRNA level was below the detection threshold in midgut tissue (Ct > 35 for both untreated control and *B. thuringiensis*-fed animals).

Fifteen hours after *E. coli* K12-injection significant differences in mRNA levels occurred in hematopoietic organs and the fat body. The hematopoietic organs increased the mRNA level of lysozyme (3.03 ± 0.44, p < 0,0001), PGRP-1A (2.63 ± 0.63, *p* = 0.0002), and scolexin A (7.18 ± 0.71, *p* < 0,0001) significantly after this systemic immune challenge (**Figure 3B**). The fat body responded with a remarkably increased scolexin A mRNA level (88.29 ± 47.65, *p* = 0.004) (**Figure 3B**). In the midgut, again IML-3 mRNA was not detectable (Ct > 35 for both saline-injected and *E. coli* K12-injected animals).

All values are given as fold expression mean ± SD; and level of significance after 2-way ANOVA with *post hoc* Tukey´s multiple comparison.

### 3.7 Lysozyme mRNA and protein in hemocytes and the hematopoietic organ

**Figure 4:**
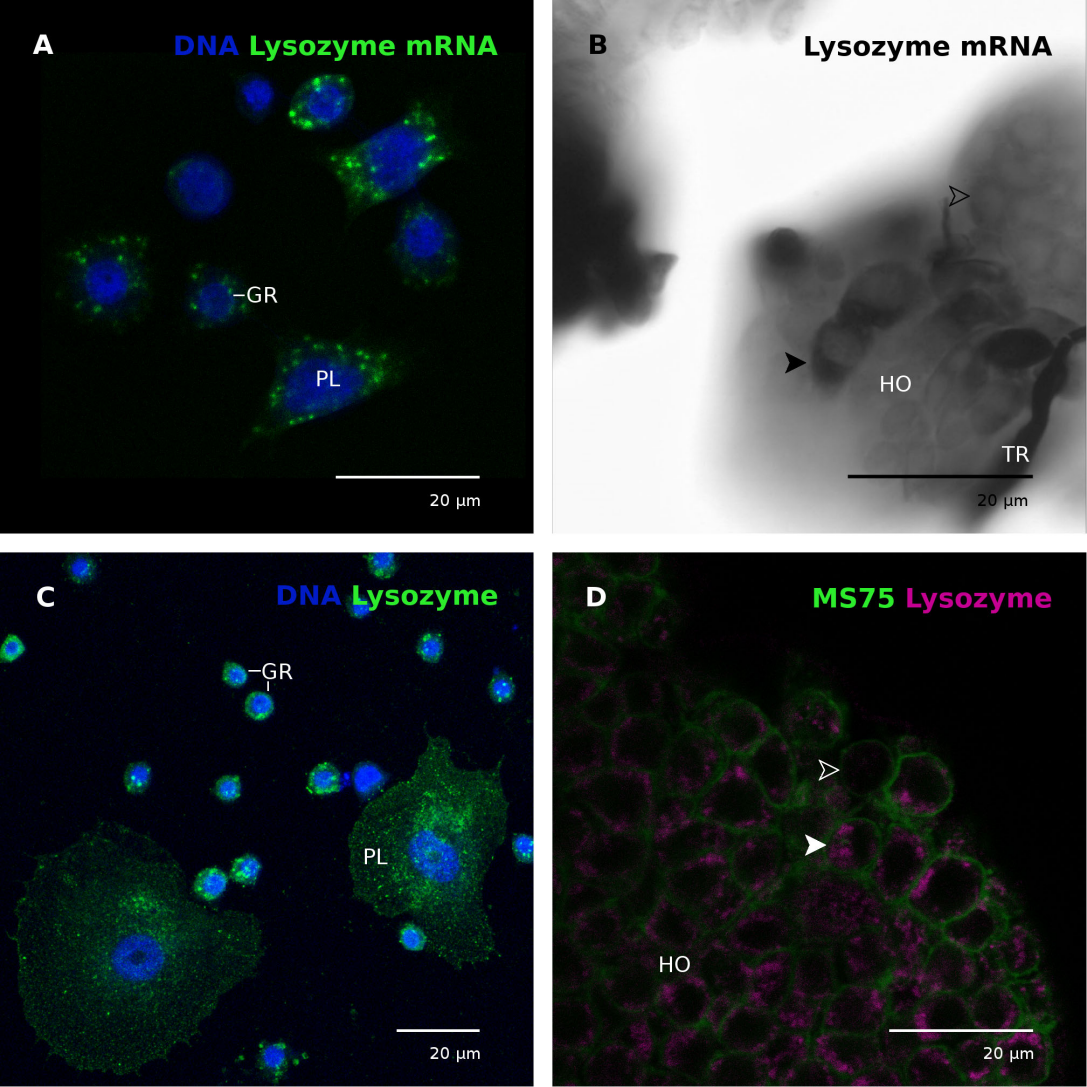
Lysozyme transcript and protein in hemocytes and the hematopoietic organ. **(A)** Plasmatocytes (PL) and granular cells (GR) contain mRNA coding for lysozyme. Nuclei labeled with DAPI (blue). The animal was injected with *E. coli* K12 15 hours prior to hemocyte collection. CLSM photomicrograph, multiple z-plane reconstruction. **(B)** Lysozyme mRNA in the hematopoietic organ (HO). Some cells exhibited stronger signals (black arrowhead) than the majority of cells (white empty arrowhead), indicating differences in the lysozyme mRNA content. The animal was wounded by cutting the horn 15 hours before hematopoietic organ isolation. **(C)** Plasmatocytes (PL) and granular cells (GR) contain granular cytoplasmic inclusions positive for lysozyme (green). Nuclei labeled with DAPI (blue). The animal was not immune-stimulated. CLSM photomicrograph, multiple z-plane reconstruction. **(D)** Most cells of the hematopoietic organ contain lysozyme (magenta) in cytoplasmic granules. A solid white arrowhead points to an exemplary lysozyme-containing cell, empty white arrowhead marks a cell without lysozyme. Cell membranes are labeled with mAb MS75 (green), indicating cell borders. The animal was not immune-stimulated. CLSM single plane photomicrograph. GR, granular cell; HO, hematopoietic organ; PL, plasmatocyte; TR, trachea.

The localization of lysozyme mRNA and lysozyme protein was determined on hemocytes and hematopoietic organs by *in situ* hybridization and antibody labeling (**Figure 4**). Based on published data (Mulnix and Dunn, 1994) to enhance the signal strength for *in situ* hybridization, some animals were wounded or injected with *E. coli* K12 15 hours before tissue extraction. The antibody labeling was conducted only on untreated animals.

mRNA coding for lysozyme was detected in circulating plasmatocytes and granular cells (**Figure 4A**). The signal appeared to be stronger in plasmatocytes than in granular cells when alkaline phosphatase was used as a reporter (**Supplementary Figure 2A**), indicating plasmatocytes as the main lysozyme synthesizing hemocyte type. The specificity of the riboprobe has been confirmed by a lack of signals when the cells were incubated with a control (i.e. non-mRNA specific) digoxigenin-labeled riboprobe (**Supplementary Figure 2**). In the hematopoietic organs only a few cells exhibited an extraordinarily strong signal for lysozyme mRNA whereas most cells showed no signal (**Figure 4B**).

The lysozyme protein is localized in circulating hemocytes in granular cytoplasmic inclusions in both plasmatocytes and granular cells (**Figure 4C**). Similarly, the protein was localized in granular cytoplasmic inclusions in most, but not all hematopoietic organ cells (**Figure 4D**).

## 4 Discussion

The insect midgut provides a tightly regulated environment for microorganisms (Engel and Moran, 2013; Caccia et al., 2016). Beneficial microorganisms are tolerated by the host while potential pathogens are recognized and repressed to keep the gut microbiota in balance. Different recognition proteins, signaling molecules, and antimicrobial peptides are involved in this regulation, and for insects many of the key players are well-characterized (Zeng et al., 2022). For example, and not surprisingly, a plethora of immunity-related genes is expressed in the *M. sexta* larval midgut (Pauchet et al., 2010; Cao et al., 2015). However, how these genes are regulated in response to gut inflammation or systemic immune challenges is mostly unknown.

### 4.1 Effects of gut infection

The *B. thuringiensis* toxin Cry1A impairs the integrity of the gut epithelium and subsequently allows gut microbiota to enter the hemocoel. The killing mechanism of *Bacillus thuringiensis* is not finally solved to date, and observations from different species contradict each other. For example in Lepidoptera, injection of *B. thuringiensis* spores is deadly even in small quantities for *M. sexta* (Johnston and Crickmore, 2009; own observations), while gut-mediated *B. thuringiensis* infection fails to kill the host in the absence of enterobacteria in the moth *Lymantria dispar* (Broderick et al., 2006).

The translocation of gut bacteria to the hemocoel is not immediate. Mason et al. (2011) found that the larval *M. sexta* gut becomes permeable for gut bacteria within 24 to 48 hours after feeding Cry1A toxin, which fits our observations that 15 hours after feeding *B. thuringiensis* no colonization of the hemolymph appeared (**Supplementary Figure 1**). To elicit a strong immune response of the midgut we infected larvae with a sublethal dose of sporulated live *B. thuringiensis* ssp. *kurstaki*. The experimental infection was not lethal within 15 hours but resulted in a total stop of food uptake within one hour and a severe and significant loss of body weight (negative growth rate, **Figure 1A**). Immune challenges decrease the growth rate of *M. sexta* (Adamo et al., 2007; Mason et al., 2011). Additionally, lysozyme activity (**Figure 2A**) as well as antimicrobial activity (**Figure 2B**) were significantly increased, and the integrity of the peritrophic membrane was impaired. These data indicate that the *B. thuringiensis* infection model was sufficient to study the effects of a severe gut inflammation. Furthermore, absence of melanotic capsules in the hemocoel and lack of CFUs from hemolymph samples (**Supplementary Figure 1**) indicate that the infection can be characterized as a severe gut infection without septic injury of the gut.

On the transcript level, the local effect was restricted to increased lysozyme expression; increased levels of mRNA coding for scolexin A and PGRP-1A were not significant. Even though the gut infection did not provoke sepsis, mRNA levels of hemocoelic tissues were altered in response to *B. thuringiensis* infection. The pattern recognition receptor PGRP-1A mRNA synthesis was upregulated in the hematopoietic organ and fat body (**Figure 3A**), and lysozyme mRNA synthesis was upregulated in hemocytes (**Figure 3A**).

An increased lysozyme mRNA synthesis in hemocytes after exposure to *B. thuringiensis* toxins is described for the cabbage looper *Trichoplusia ni*, too (Tamez-Guerra et al., 2008). In *D. melanogaster* larvae, oral application of the entomopathogenic bacteria *Erwinia carotovora* ssp. *carotovora* strain 15 (Basset et al., 2000) or *Pseudomonas entomophila* (Vodovar et al., 2005) led to the increased expression of antimicrobial peptides in gut tissue (local contact zone) as well as in the fat body, indicating cross-talk between the gut and the hemocoel. These data confirm the involvement of both local tissue and tissue not in direct contact with the gut pathogens to react to the gut-borne infection before a severe invasion of the hemocoel occurs.

How the infection is signaled from the gut epithelium to the hemocoel is unknown. Two principal ways how a gut-borne infection may be sensed and the signal transmitted to the hemocoel in insects are known. (1) Microorganisms or immunogenic molecules may cross the barrier of the infected gut as proposed for PGN fragments (Zaidman-Rémy et al., 2006), or (2) an active signaling process of the gut epithelium is involved. As an example of the latter, it was shown in *D. melanogaster* that nitric oxide (NO) triggers AMP synthesis in the fat body via the Imd-pathway after the gut comes in contact with pathogenic bacteria (Foley and O'Farrell, 2003). Nitric oxide plays also a role in the local response of the midgut to enterobacteria *Photorhabdus luminescens* in *M. sexta*, leading to a reduced number of pathogens in the hemolymph and the gut when upregulated (Eleftherianos et al., 2009). This dual role as an antimicrobial substance and signaling molecule is a promising model to explain the transmission of the infection signal sent by the inflamed gut to the hemocoelic organs.

However, it is imaginable that sensing an unusually high load of microorganisms in the midgut may trigger a preemptive immune activation. To test this hypothesis, we administered live *E. coli* K12 *per os* and determined changes in the immune gene mRNA levels. Only a slight increase of PGRP-1A mRNA level occurred, and scolexin A mRNA expression of the midgut was decreased (**Supplementary Figure 3**). In another set of experiments, *E. coli* supplemented artificial diet even increased the growth rate of younger (L2/L3) instars (Trenczek, own observation). This indicates that an unusually high abundance of microbes alone may not be sufficient to trigger a (preventive) immune response, but that increased immune activity in the midgut and hemocoel occurs only in response to a true infection or inflammation.

### 4.2 Effects of bacteria injection

To simulate a systemic infection, e.g. as it might occur after gut leakage provoked by *B. thuringiensis* toxins, or when microorganisms enter hemocoel through an epidermal wound, we injected live *E. coli* K12 into the hemocoel. Injection of *B. thuringiensis* ssp. kurstaki through a cuticular wound turned out to be fatal for *M. sexta* larvae (data not shown; cf. Johnston and Crickmore, 2009) and was therefore not suitable for our experiments. *E. coli* is a typical gut commensal and shares characteristics of its cell wall composition (DAP-type PGN) with *B. thuringiensis* (Kingan and Ensign, 1968). The injection procedure itself (saline-injection) lowered the larval growth rate while the lytic and antibacterial activity of the hemolymph did not increase. Hence, we conclude that in our infection model, wounding and saline-injection lead to a physiological response but only minor immunological changes (as measured in activity changes).

The injection of *E. coli* K12 resulted in the formation of numerous melanotic capsules (noduli) found throughout the body cavity. Moreover, bacteria were recovered in high numbers from the hemolymph 15 hours post injection (**Supplementary Figure 1**). Additionally, the lysozyme activity and the antimicrobial activity of the hemolymph increased significantly (**Figures 2A, B**). On the other hand, neither the body weight growth rate (**Figure 1**) nor the integrity of the peritrophic membrane was affected, as opposed to *B. thuringiensis-fed* animals. Taken together, the injection of live *E. coli* K12 provoked a similar increase in both the lytic activity and the antimicrobial activity of the hemolymph compared to the gut infection, while the peritrophic membrane and growth rate remained largely unaffected.

In the hematopoietic organ, mRNA coding for lysozyme, PGRP-1A, and scolexin A was increased, the hemocytes increased lysozyme mRNA synthesis, and an elevated level of mRNA coding for lysozyme and scolexin A was detected in the fat body, the latter being dramatically increased compared to saline-injected controls (**Figure 3B**). Most importantly, other than in *B. thuringiensis*-infected animals, the midgut did not alter the mRNA expression of the observed immune-related genes significantly.

### 4.3 Parallels between gut and systemic infection

The midgut did not react with significant mRNA level alterations on *E. coli* K12-injection into the hemocoel, while it did on a local infection of the midgut itself. On the other hand, cells located within the hemocoel respond to both midgut and systemic infections. Therefore, we propose the activation of organs in the hemocoel in response to a gut-borne infection before direct contact of the hemocoel with the pathogen occurs, which is mediated through a yet not analyzed pathway. Either immunogenic molecular patterns cross the gut epithelium and elicit the immune response of the fat body, the hemocytes, and the hematopoietic organs or signaling takes place through mediators, probably released by the gut epithelium, activating these organs. Conversely, an infection of the hemocoel mimicked by injection of *E. coli* K12 did not alter the mRNA level of the observed genes in the midgut. Data from other insects, however, are contrary to our findings. In the common bedbug *Cimex lectularius* (Heteroptera, Cimicidae) for example, the expression of defensin genes of the gut tissue was increased in response to a hemocoelic injected bacterial mixture (Meraj et al., 2021). Similarly, in the mosquito *Anopheles stephensi* (Diptera, Culicidae), an injection of *E. coli* K12 into the hemocoel increased AMP expression in the midgut (Das De et al., 2018). Since both species are hematophagous, with often long starving periods between blood meals, their gut defense may play a more important role in systemic infections than in the herbivorous, constantly feeding *M. sexta* larva. We do not state that the midgut of *M. sexta* does not react at all to a systemic immune threat, e.g. by increased AMP expression as was shown for larvae inoculated with a mixture of heat-killed bacteria and fungi mixture (McMillan and Adamo, 2020) but rather suggest to compare the gut response against systemic infections in a broad range of insect species from different ecological niches. Especially detritivores such as many Diptera larvae, feeding on a diet with a high load and diversity of microbes, and parasites that may encounter highly adapted pathogens and parasites through food uptake, e.g. mosquitos, can be expected to exhibit stronger involvement of the gut immune system to both gut and systemic infections as well as a stronger anticipation of systemic immune events after sensing gut mediated infections.

### 4.4 Circulating hemocytes and the hematopoietic organ synthesize andstore lysozyme

The hemocytes of insects possess lysozyme-containing phagosomes (Zachary and Hoffmann, 1984) and secrete lysozyme (Anderson and Cook, 1979). Lysozyme may therefore be a marker for immune-competent hemocytes. Interestingly, we found lysozyme to be expressed in granular cells and plasmatocytes, the most abundant hemocyte types in *M. sexta* (**Figures 4A, C**), both cell types involved in phagocytosis and encapsulation (Horohov and Dunn, 1983; Levin et al., 2005; Ling and Yu, 2006). The hematopoietic organ, however, forms and releases plasmatocytes (Nardi et al., 2003; von Bredow et al., 2021). Because some of the cells of the hematopoietic organ contain a higher amount of lysozyme mRNA these cells likely represent mature plasmatocytes (**Figure 4B**). Similarly, not all cells of the organ contain lysozyme protein (**Figure 4C**). Upregulation of lysozyme expression in response to a systemic infection indicates either a putative role of the hematopoietic organs in the humoral defense by secreting lysozyme or an increased hemocyte maturation, forming more immune competent cells (plasmatocytes) in response to an infection.

Hematopoietic organ cell lysates exhibited lytic activity (data not shown), further supporting the idea of the role of the organs in humoral immune activity.

### 4.5 The hematopoietic organ reacts to infection

Research on the roles of hematopoietic tissues in insect immunity has been restricted mostly to increased proliferation (e.g. Duressa et al., 2015), differentiation of specific hemocyte types (e.g. Lanot et al., 2001), or its phagocytic properties (e.g. Nutting, 1951; Hoffmann, 1970). However, to our knowledge, no studies concerning changes in the expression of distinct immunity-related molecules, or involvement in humoral immune responses were conducted before for lepidopteran hematopoietic organs. Hence, we present the first description of differential expression of immune molecules in the hematopoietic organ of *M. sexta*, and show that the mRNA levels of lysozyme, PGRP-1A, and scolexin A alter when systemic infections occur and that this tissue reacts by increased PGRP-1A expression in response to gut infection.

Since the hematopoietic organs form plasmatocytes (Nardi et al., 2003; von Bredow et al., 2021), the expression of lysozyme within a subset of hematopoietic organ cells may be indicative of mature plasmatocytes contained by the organs. The increase in immune-related gene expression could therefore be due to an increased differentiation of the hematopoietic organ cells to mature plasmatocytes. Moreover, we cannot rule out that the organ itself plays a role in the humoral defense, being a minor source of receptors and effectors secreted to the hemolymph. However, a direct comparison of the reaction of hemocytes and the hematopoietic organ is difficult, since the hemocytes in circulation comprise a mixture of granular cells, plasmatocytes, spherule cells, oenocytoids, and prohemocytes whereas the hematopoietic organs do not contain granular cells, spherule cells or oenocytoids (Nardi et al., 2003; von Bredow et al., 2021).

## 5 Conclusions and outlook

The intestine is the first contact zone for ingested pathogens and at the same time harbors beneficial microbes, which requires a fine-tuned system of immune recognition and response. The gut microbiota and the gut immune system influence each other, and imbalances in the gut immune system – and microbiota – are symptoms and likely causes of many human diseases, including chronic inflammatory diseases (Abraham and Medzhitov, 2011; Jiao et al., 2020).

**Figure 5:**
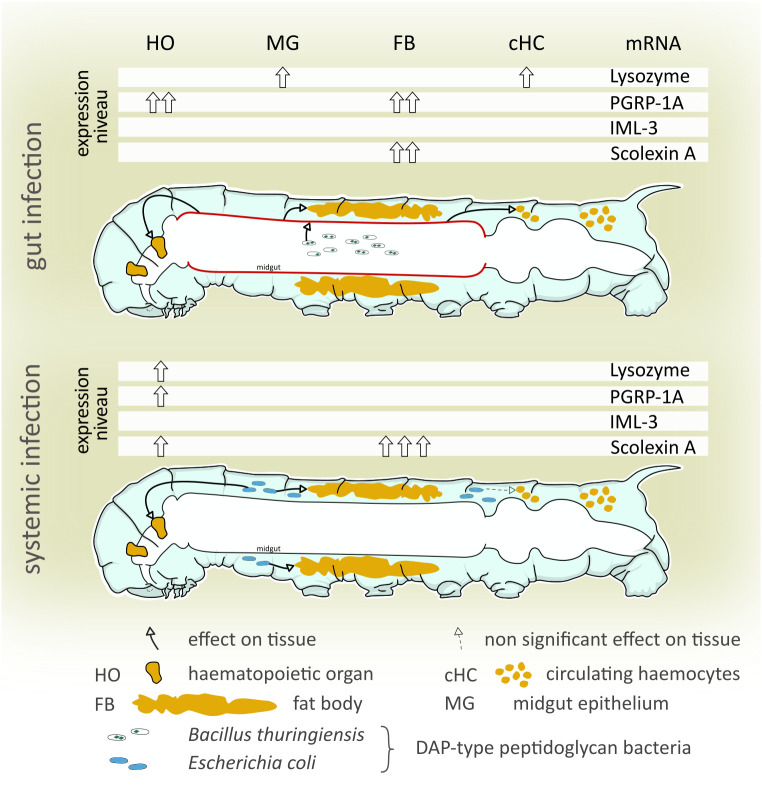
Summary of the differential immune-related transcript levels in response to gut and systemic infection. We examined differences and similarities of the immune response to gut-borne and systemic immune challenges in the tobacco hornworm (*Manduca sexta*). Both gut-borne and systemic infections increased peptidoglycanolytic and antibacterial activity of the hemolymph, and immune-related transcript levels differed highly between treatments and tissues examined. A non-bacteremic gut infection (*B. thuringiensis*) increases local (midgut) and systemic (hematopoietic organs, hemocytes, and fat body) immune-related gene levels. Systemic immune stimulation (hemocoelic injection of *E. coli* K12) significantly increased immune gene transcript levels in hematopoietic organs and the fat body, but not the midgut. This indicates that (i) even non-bacteremic gut-borne immune challenges induce a systemic immune response while (ii) a systemic immune challenge lacks involvement of the midgut in a systemic immune response.

Insect intestinal immunity and vertebrate intestinal immunity share many similarities like evolutionary conserved immune-related receptors and effectors as well as phagocytic cells (Bergman et al., 2017). Gut-associated lymphoid tissues (GALTs) of vertebrates harbor immune cells that serve as sentinels and regulators of the gut. In *D. melanogaster*, gut-associated phagocytes may serve a similar immune protective and regulatory function (Zaidman-Rémy et al., 2012), indicating a close association of the gut and internal immune cells in the insect. In the present paper, we demonstrate that an infection of the midgut results in a local response of the midgut epithelium and systemic response of tissues in the hemocoel even when no bacteremia occurs (**Figure 5**). On the other hand, an infection in the hemocoel affected mRNA expression of the observed genes of the hemocoelic tissues but not of the midgut (**Figure 5**). Therefore, we conclude that cross-talk between midgut and organs in the hemocoel takes place, preparing the latter for an imminent invasion of the hemocoel. How the signal is transduced is unclear, both pathogen fragments crossing the gut border and intrinsic signals released by the gut cells are imaginable to take into account for a systemic immune response. Experimental evidence for this transmission should be provided by future experiments.

For the hematopoietic organs, we showed that an increased mRNA synthesis of lysozyme, PGRP-1A, and scolexin A was induced by systemic infection (**Figure 5**), suggesting either an increased differentiation to mature, immune-competent hemocytes taking place within the organ or that the cells of the hematopoietic organs increase the synthesis of immune-related molecules independent from hemocyte differentiation. The finding that lysozyme is synthesized in cells of the hematopoietic organ is further evidence of the role of the hematopoietic organ in the immune response beyond the production and release of hemocytes. Future analyses should address the question if the hematopoietic organ does produce and release immune-related receptors and effectors, and consequently might be an additional source for immune molecules secreted into the hemolymph complementing the fat body and circulating hemocytes.

## Data availability statement

The original contributions presented in the study are included in the article/**Supplementary Material**, further inquiries can be directed to the corresponding author/s.

## Author contributions

YvB: Conceptualization, Formal Analysis, Funding acquisition, Investigation, Methodology, Visualization, Writing – original draft, Writing – review & editing, Project administration. PP: Writing – review & editing, Formal Analysis, Visualization, Writing – original draft. JD: Formal Analysis, Funding acquisition, Investigation, Methodology, Writing – review & editing. FS: Funding acquisition, Methodology, Writing – review & editing. TT: Funding acquisition, Writing – review & editing, Conceptualization, Project administration, Resources. MB: Funding acquisition, Writing – review & editing, Project administration, Resources. CvB: Conceptualization, Funding acquisition, Project administration, Formal Analysis, Investigation, Methodology, Visualization, Writing – original draft, Writing – review & editing.
