# Supplementary material for: Differential expression of immunity-related genes in larval Manduca sexta tissues in response to gut and systemic infection
Source: Front Cell Infect Microbiol. 2023 Oct 11;13:1258142. doi: 10.3389/fcimb.2023.1258142 (PMC10603244; doi:10.3389/fcimb.2023.1258142)
Supplement: Supplementary file 1 [file DataSheet_1.pdf]

Differential expression of immunity-related genes in larval *Manduca sexta* tissues in response to gut and systemic infection

Yvette M. von Bredow[1], Petra Prochazkova[2], Jiri Dvorak[2], Frantisek Skanta[2], Tina E. Trenczek[1], Martin Bilej[2], Christoph-Rüdiger von Bredow[1,3*]

[1] Institute of Zoology and Developmental Biology, Justus-Liebig University Gießen, Gießen, Germany

[2] Laboratory of Cellular and Molecular Immunology, Institute of Microbiology of the Czech Academy of Sciences, Prague, Czechia

[3] Applied Zoology, Department of Biology, Technische Universität Dresden, Dresden, Germany

***Correspondence:**

Christoph-Rüdiger von Bredow

christoph.von.bredow@mail.de

Supplementary material

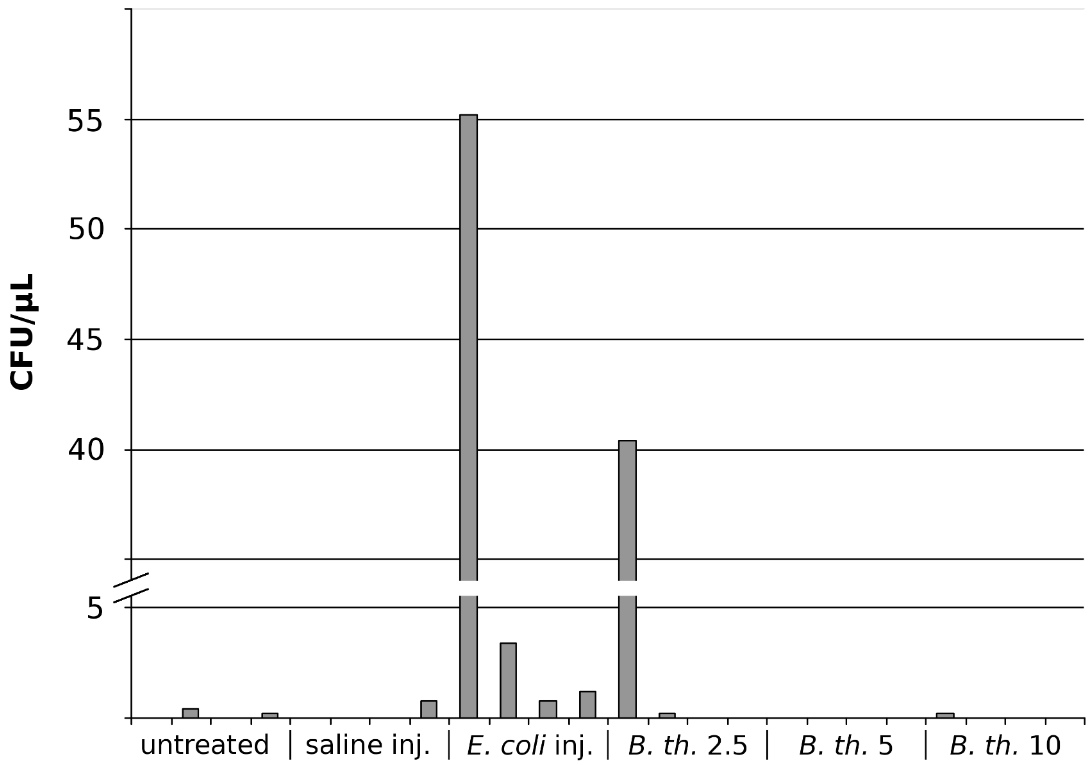

Supplementary figure 1: Bacteria recovery from hemolymph of different treatments. The number of bacteria was determined as colony-forming units (CFUs) per µl hemolymph. CFUs of $n = 4$ animals were determined. Treatments: saline injection (saline inj.), *E. coli* K12-injection (*E. coli* inj.), *B. thuringiensis* ssp. *kurstaki*-fed, three different concentrations: $2.5*10^6$ (*B. th.* 2.5), $5*10^6$ (*B. th.* 5), and $1*10^7$ (*B. th.* 10) spores per diet cube ($1cm^3$).

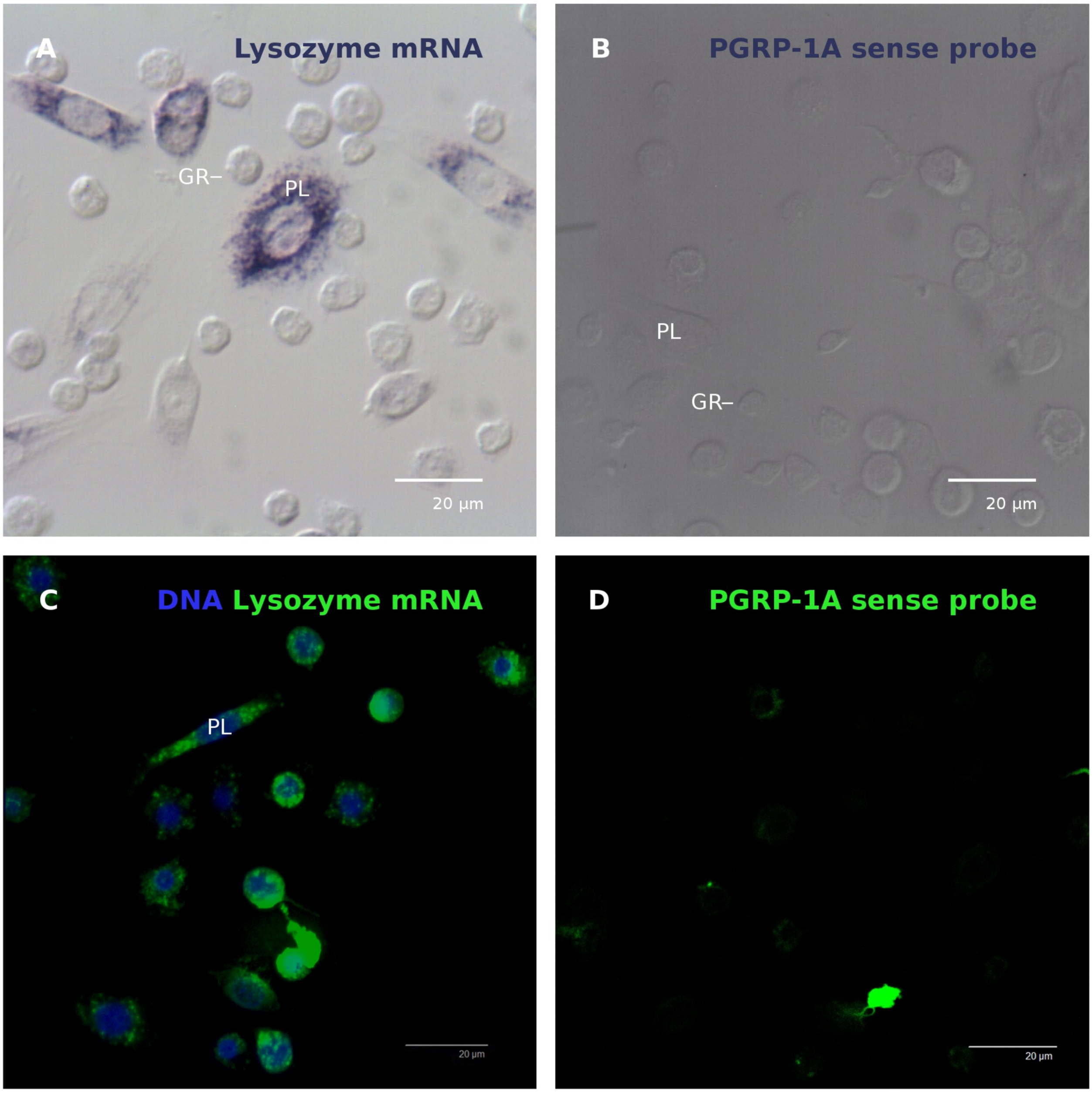

Supplementary figure 2: mRNA *in situ* hybridization with lysozyme specific riboprobes (A, C) and corresponding negative controls with sense riboprobes against PGRP-1A (B, D). Animals were wounded by removing the abdominal horn 15 hours before hemocyte extraction (A, B) or injected with *E. coli* K12 15 h prior to hemocyte extraction. Lysozyme-specific antisense riboprobes specifically label cytoplasm of plasmatocytes (PL) and granular cells (GR) in both ISH (A) and FISH (C) samples. Note absence of specific signals in both ISH (B) and FISH (D) samples incubated with digoxigenin-labelled sense riboprobes. Fluorescence signals in D are non-cellular artifacts.

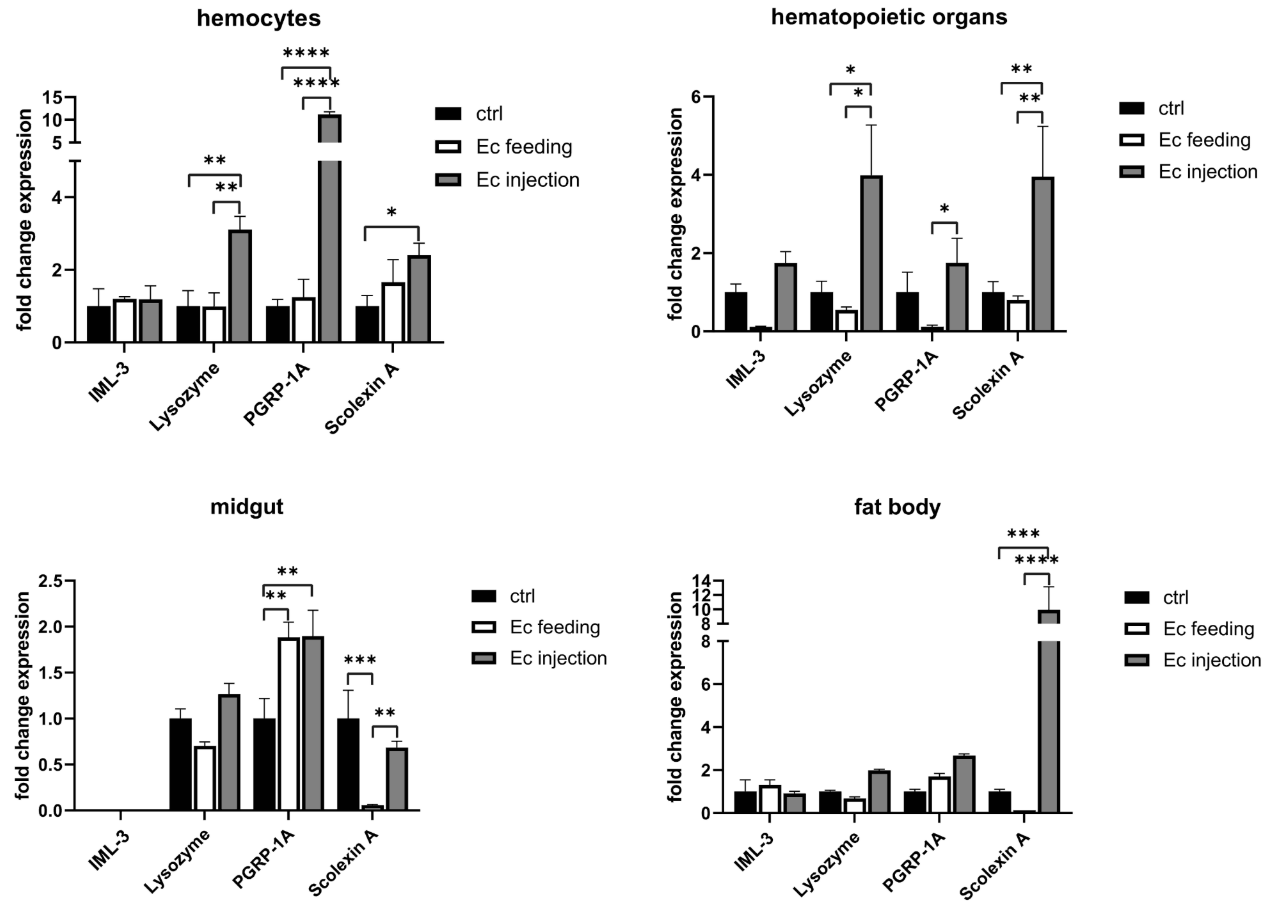

Supplementary figure 3: Effects of dietary uptake of *E. coli* K12 on tissue specific immune related mRNA levels. Animals were fed with artificial diet containing 3.6×10^7 live *E. coli* K12 for 15 hours (six $1cm^3$ diet cubes containing 6×10^6 bacteria per cube). mRNA levels were compared to untreated control (ctrl) and *E. coli* K12-injected (Ec injection) cohorts. Asterisks mark significant differences (* $p \leq 0.05$; ** $p \leq 0.01$, *** $p \leq 0.001$, **** $p \leq 0.0001$; two-way ANOVA with *post hoc* Tukey´s multiple comparison). Only PGRP-1A and Scolexin A mRNA of the midgut tissue was significantly altered in response to ingested *E. coli* K12. Abbreviations: ctrl, untreated control; Ec feeding, *E. coli* K12-fed; Ec injection, *E. coli* K12-injected.
